# MADFU: An Improved Malicious Application Detection Method Based on Features Uncertainty

**DOI:** 10.3390/e22070792

**Published:** 2020-07-20

**Authors:** Hongli Yuan, Yongchuan Tang

**Affiliations:** 1 Institute of information engineering, Anhui Xinhua University, Hefei 230088, China; 2 School of Big Data and Software Engineering, Chongqing University, Chongqing 401331, China

**Keywords:** Android app, detection, MCMC, uncertainty, machine learning

## Abstract

Millions of Android applications (apps) are widely used today. Meanwhile, the number of malicious apps has increased exponentially. Currently, there are many security detection technologies for Android apps, such as static detection and dynamic detection. However, the uncertainty of the features in detection is not considered sufficiently in these technologies. Permissions play an important role in the security detection of Android apps. In this paper, a malicious application detection model based on features uncertainty (MADFU) is proposed. MADFU uses logistic regression function to describe the input (permissions) and output (labels) relationship. Moreover, it uses the Markov chain Monte Carlo (MCMC) algorithm to solve features’ uncertainty. After experimenting with 2037 samples, for malware detection, MADFU achieves an accuracy of up to 95.5%, and the false positive rate (FPR) is 1.2%. MADFU’s Android app detection accuracy is higher than the accuracy of directly using 24 dangerous permission. The results also indicate that the method for an unknown/new sample’s detection accuracy is 92.7%. Compared to other state-of-the-art approaches, the proposed method is more effective and efficient, by detecting malware.

## 1. Introduction

There are billions of mobile Internet users worldwide currently. Moreover, Android has become the most popular mobile operating system. Millions of Android apps are installed by mobile devices. Meanwhile, a large number of Android malicious apps are constantly appearing [1]. AppBrain [2] shows that, by the end of February 2019, there were more than 2.5 million apps available on Google Play. Notably, 12% of them are considered as low-quality or potentially harmful apps. Until 30 June 2019, the 360 Security Center intercepted more than 49.663 million mobile phone malicious samples. Nearly 18,000 new mobile phone malicious samples are intercepted every day [3]. These malicious apps have brought many inconveniences (including privacy theft, remote control, tariff consumption, malicious deduction and rogue behavior) to users.

In order to remove malicious apps and low-quality apps from the application market, a large number of malicious app detection technologies have been applied, such as static detection [4,5,6,7] and dynamic detection [8,9,10]. Static detection does not need to run the app. It analyzes the files in the APK package to determine whether the app is benign or malicious [6]. Static detection consumes less time and resources. However, static detection technology is difficult to detect obfuscated or repackaged malicious Apps [1]. In contrast, dynamic analysis attempts to identify malicious behavior after deploying and executing applications on an emulator or real device [9]. However, dynamic analysis consumes more of the Android Operating System (OS) and takes longer. Moreover, there is no guarantee that all actions will be executed and detected [11].

Whether it is static analysis or dynamic analysis, the machine learning method is widely used in the classification process [12,13,14,15,16,17]. It is extremely important to construct features during classification with machine learning. The quality of the selected features determines the performance of the detection. K. Zhao et al. [18] presented a Feature Extraction and Selection Tool (FEST). FEST has nearly 98% accuracy and recall, with only 2% false alarms. D.F. Li et al. [19] presented an automatic malware family detection tool (FgDetector). FgDetector can extract features from an Android application and convert it into a low-dimensional feature vector for training the detection model to detect whether an application is malicious or not. Mahmood D. [20] proposed the ERS model. The model calculates the risk of permissions through entropy. The ERS values are then used for machine learning classification.

Although rapid progress has been made in extracting features from applications to detect malicious applications, there are still some open issues. Firstly, these Android app classification methods, based on machine learning, all discard the analysis of features uncertainty, but features uncertainty plays a vital role in malicious detection. Secondly, the extraction of features may be time-consuming, due to the increasing size and highly complicated behaviors of an APK, resulting in noneffective detection. Our paper aims to address these two problems. Based on the idea of the permission risk values proposed by ERS Model, the uncertain value of permission is calculated by the Markov chain Monte Carlo (MCMC) algorithm and applied to malicious detection.

In this paper, a new method, named a malicious application detection model, based on features uncertainty (MADFU) is presented. Firstly, MADFU extracted 24 dangerous permissions from the app. Secondly, the MCMC algorithm is used to calculate the permission uncertainty value in the detection of a malicious app. Thirdly, based on the uncertainty value, MADFU removes permissions with small contribution values, and keeps permissions with large contribution values. Finally, permissions with large contributions are used for classification.

The contributions of our paper are the following.

- MADFU uses the MCMC algorithm to calculate the uncertainty of the permission’s features in malicious detection. The results show that ignoring uncertainty can lead to false positives in the analysis.
- MADFU removes the uncertainty permissions and uses purified permissions to classify through machine learning.
- We used 2037 samples to verify the model. MADFU has good Accuracy for both known samples and new samples. Meanwhile, MADFU reduces memory consumption and classification times.

The rest of this paper is organized as follows. A brief introduction to the characteristics of Android permissions and MCMC is given in Section 2. Section 3 introduces the methodology of this paper. In Section 4 is an evaluation and a discussion. The last section concludes this paper.

## 2. Background

### 2.1. Android Permission

In order to protect users’ information security, the Android app uses a permission mechanism to access the user’s sensitive information [1]. When an app is installed, it will provide users with a list of permissions for the app. When the user accepts these permissions, the application is installed on the device. If an app wants to use a resource, they need to declare it in the manifest.XML file. An undeclared permission Android system cannot be used [11].

There are 135 Android app permissions [11]. App permission is divided into four types according to security; namely normal, dangerous, signature, and signature/system. Low-Level permissions, including normal and dangerous levels, are authorized as soon as an app is used. Signature level and signature/system level permissions are known as advanced permissions [1]. Among them, Google proposed 24 dangerous permissions. Including SEND_SMS, CALL_PHONE, CAMERA, etc.—these permissions allow one to access users’ sensitive information.

There exist many approaches for detecting Android apps by extracting permissions. Some methods only use permissions to classify the Android app [11,12,21], while other methods combine other features (such as API, CFG, etc.) to classify [22,23,24]. Wang et al. [12] analyzed the risks of individual permissions and collaborative permissions. They ranked the individual permissions with respect to their risks. Sarma et al. [21] used both the permissions that the app requested, and permissions requested by other apps in the same category. Peiravian et al. [22] chose not only the requested permissions, but also permissions based on the app’s API calls, which are called used permissions.

Android permission is the most used and effective static feature. It is because attackers usually achieve their goals through permission [1]. Therefore, MADFU uses requested permissions and declared permissions to classify Android apps.

### 2.2. MCMC

In machine learning, complex probabilistic models usually need to calculate complex high-dimensional integrals [24]. For example, for a classification task, we need to predict the class of the instance. Suppose $\int p\left(y_{*}|x_{*},\theta \right)p(\theta |\tau )d\theta$ is a prediction model, where $x_{*}$ represents the instance, $y_{*}$ represents the class, τ represents the data, $p\left(y_{*}|x_{*},\theta \right)$ is the likelihood function and p(θ|τ) is the posterior distribution. When the probability model becomes complicated, this integral is difficult to handle. Markov chain Monte Carlo (MCMC) is suitable for approximate integration. MCMC is a powerful framework, which is widely used to deal with complex and intractable probabilistic models [24].

The Markov chain Monte Carlo method (MCMC) introduces the Markov process into Monte Carlo simulation [25]. It implements the dynamic simulation of random sampling [26]. It makes up for the defect that the traditional Monte Carlo method can only simulate statically. The basic idea is to construct a Markov chain. Make the stable distribution of the Markov chain as the posterior distribution of the parameters to be estimated [27]. Monte Carlo integration is performed on samples with posterior distribution [28].

When using the MCMC method, the construction of the Markov chain transfer core is very important. Different transfer and construction methods will produce different MCMC sampling results. There are two main MCMC methods in common use: the Gibbs algorithm [29,30] and the Metropolis–Hastings algorithm [31,32]. The Gibbs algorithm is a special Metropolis algorithm [33,34].

## 3. MADFU Model

**Figure 1:**
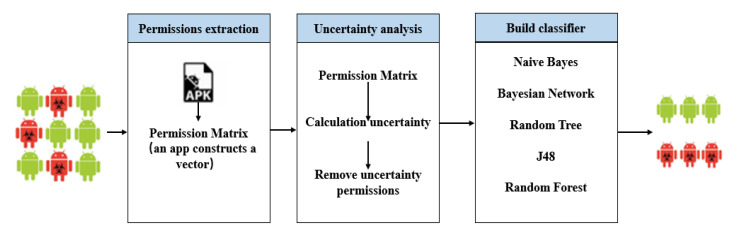
Overview of a malicious application detection model based on features uncertainty (MADFU).

This section mainly introduces the MADFU model proposed in this paper. The MADFU model consists of three parts: Samples collection and features extraction, features’ uncertainty analysis and machine learning. As shown in Figure 1.

### 3.1. Permissions Extraction

**Figure 2:**
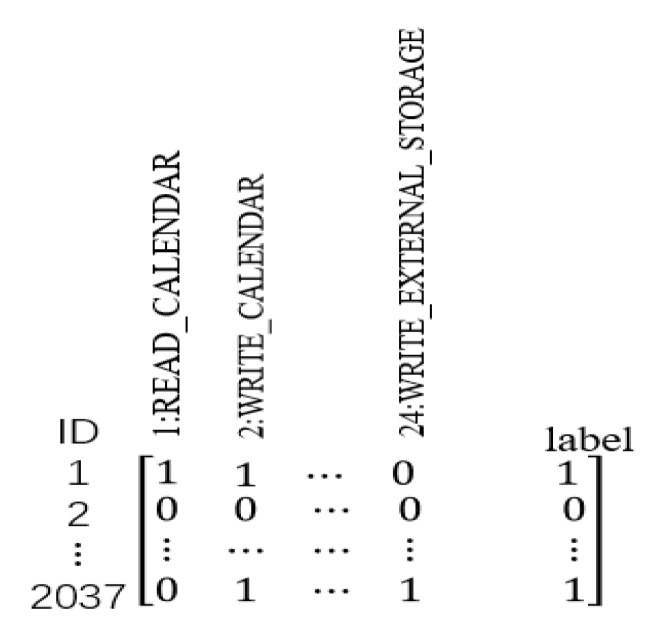
Lists of permissions.

The MADFU model uses a python program to crawl the benign app in the website (such as Wandoujia, Huawei store), and downloads the malicious Apps in the drebin dataset [35]. The APK file is decompiled by APKTool. The decompiled files include folders such as manifest files and smali files. This model uses the Python program to extract the Android Manifest.xml file and class files containing the permission statement. The 24 dangerous permissions declared by Android in Google are extracted in the Android Manifest.xml file. The extracted permission matrix is shown in Figure 2.

### 3.2. Uncertainty Analysis

In the MCMC calculation, a suitable function needs to be selected for the sampled posterior distribution model. Logistic regression [36] is a machine learning method used to solve binary classification (0 or 1) problems, and is used to estimate the likelihood of the object. In order to calculate the uncertainty of the Android app’s security detection process, this model uses a logistic regression model to express the relationship between app characteristics and app labels (benign or malicious).

The logistic regression function is defined as follows:

(1) $$y=\frac{1}{1+e^{-\left(\beta x+\alpha \right)}}$$

where *y* is the classified labels (benign or malicious), *x* is the input value of the feature, *β* is the weight of the parameters in the model, *α* is the measurement noise. Moreover, *α* and *β* simulate their values through MCMC. Logistic regression assumes that the dependent variable *y* follows the Bernoulli distribution.

(2) $$y\sim Bernoulli\frac{1}{1+e^{-\left(\beta x+\alpha \right)}}$$

In order to extract the random values of *α* and *β*, it is necessary to assume a prior distribution for each coefficient. It is assumed that *α* and *β* are expected to follow a normal distribution.

(3) $$\alpha \;\sim \;N\left(\mu_{i},\delta_{i}^{2}\right)$$

(4) $$\beta \;\sim \;N\left(\mu_{i},\delta_{i}^{2}\right)$$

**Figure 3:**
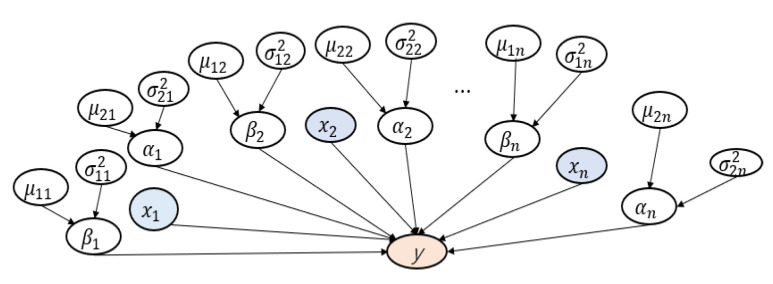
Graphical Model for Android Permission Analysis.

In summary, the parameter relationship in the logistic regression model is shown as Figure 3.

Using the Metropolis–Hastings algorithm to sample the posterior distribution of *α* and *β*, the algorithm (Algorithm 1) is shown as below.


**Algorithm 1**
1: **Input:** 2:  P (x): initial probability distribution; 3:  Q: state transition matrix; the corresponding element is q(j|i) 4: **Output:** 5:  X: sample sequence 6:   Step1: Initialize the Markov chain state X_0=x_0 7:   Step2: for time=0, 1, 2, …, do 8:     X_t=x_t sampling y~q(x|x_t) 9:     Sampling form uniform distribution: u~uniform [0,1] 10:      if u<α(y│x_t)=min{(P(y)q(x_t│y))/(P(x_t)q(y|x_t)),1} then 11:       x_(t+1)=y 12:      else x_(t+1)=x_t 13:   step3: return X

Through MCMC sampling, the 95% confidence interval of the highest probability density interval of beta can be calculated. The larger the interval, the greater the uncertainty value of the permission, and the less suitable it is for malicious detection. On the contrary, it is more suitable for malicious detection.

### 3.3. Machine Learning Classification

The datasets are trained and tested by naive Bayes (NB), Bayesian network (BN), J48, random tree (RT) and random forest (RF) machine learning classification algorithms. The test method uses a 10-fold cross-validation test method. 

We evaluate our model with five metrics: false positive rate (FPR), accuracy, F-measure, ROC and AUC. These metrics are shown in Appendix A.

## 4. Evaluation

This section mainly introduces the experimental results and discusses the results.

### 4.1. Datasets

In the experiment, 2037 APKs (1058 benign samples and 979 malware samples) are used for classification. Overall, 1058 samples were used to analyze the detection performance of MADFU. Moreover, 892 samples were used to analyze the detection ability of MADFU on unknown samples. In total, 2037 samples rang from October 2012 to June 2018. All these samples are scanned and confirmed by malware detection tools (VirusTotal [37] and Virscan [38]).

### 4.2. Experimental Methods

In order to analyze the uncertainty of dangerous permissions, MADFU uses the Python language and the data science package (Theano [39] and PyMC3 [40]), to implement the Metropolis algorithm. We run our Metropolis algorithm on an Intel Core i7-7700HQ fourth-generation processor with 8 cores clocked at 3.5 GHz, and with 32 GB of on-board memory. The PyMC3 code is shown in Appendix B.

**Figure 4:**
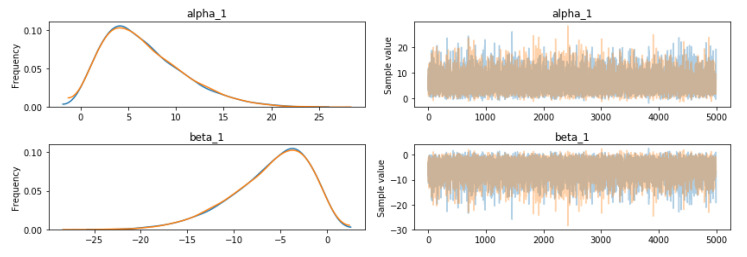
Sampled values of alpha (*α*) and beta (*β*) for READ_PHONE_STATE (left is Autocorrplot, right is Traceplot).

For the MCMC run, MADFU selected 5000 samples for analysis, which ensures that the model converges before sampling. The traceplot and autocorrplot of the alpha (*α*) and beta (*β*) parameters for READ_PHONE_STATE are shown in Figure 4.

When using MCMC, the initially generated values are often inaccurate. After the Markov chain converges, the generated parameters are used to model the values. We used 10,000 samples to calculate. The previous 50% sample was abandoned.

### 4.3. The Uncertainty of Permissions

**Figure 5:**
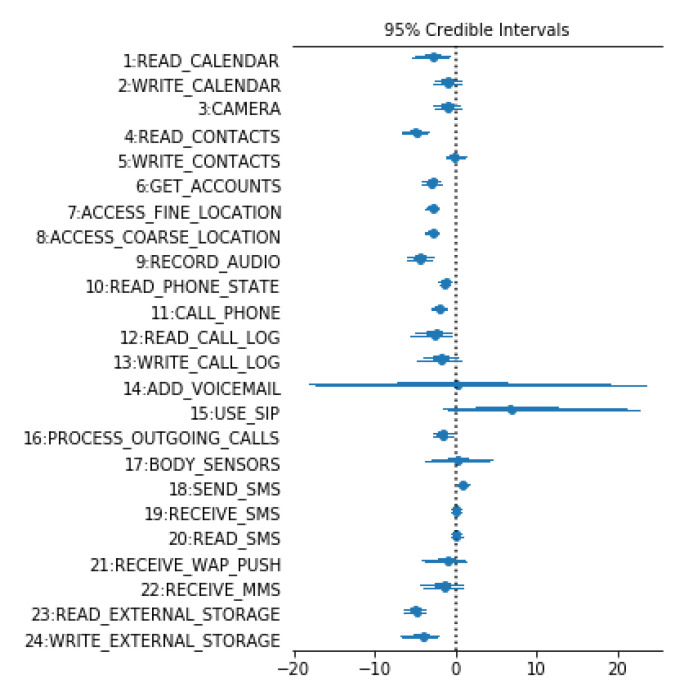
Forest Plot of Interesting β Values and their Associated Uncertainties. (Right is benign, left is malicious.)

After MCMC runs, and the posterior probability of all parameters is calculated, the density function set of different parameters in MADFU model are obtained, since it is impossible to display a detailed view of the posterior probabilities of all parameters of the model. Thus, the forest plot is used to show the uncertainty of β for 24 dangerous permissions, as shown in Figure 5.

An interesting result can be seen from Figure 5; *β* of the ‘SEND_SMS’ is on the right side of the benign label. This means that although this permission is a dangerous permission, it is used by benign Apps more often. This is different from what was previously assumed. After analysis, many apps are registered with a phone number when they are used. A registration verification code needs to be sent during registration. Benign apps are used more often than malicious apps. In contrast, the probability that READ_CALENDAR is used in malicious apps is much higher than in benign apps. READ_CONTACTS and GET_ACCOUNTS, etc. are similar to READ_CALENDAR. Because permissions which are frequently used in malware (or benign apps) and rarely used in benign apps (or malware) are more important when distinguishing malware from benign apps. So, these permissions are used to detect malicious apps.

The *β* value of ‘ADD_VOICEMAIL’ spans very large. This means that ‘ADD_VOICEMAIL’ has a high degree of uncertainty in Android app detection. So, the permission ‘ADD_VOICEMAIL’ cannot be accurately distinguished between benign apps and malicious apps. Using ‘ADD_VOICEMAIL’ to classify Android apps is useless.

It can also be seen from Figure 5 that the uncertainty of the permission for ‘WRITE-CONTACTS’, ‘RECEIVE_SMS’ and ‘READ_SMS’ are small. However, their means are close to zero. This means that the three of them are used similarly in malicious apps and benign apps. Therefore, it is not meaningful to use them for classification. The mean value of the ‘BODY_SENSORS’ is close to zero, with a high degree of uncertainty. So, the ‘BODY_SENSORS’ is not suitable for malicious detection.

The value of ‘USE_SIP’ spans more on benign apps, but its uncertain value exceeds 20, and a portion of it spans on malicious apps. Therefore, it makes little contribution to the detection of Android apps.

In summary, in MADFU, we removed 6 permissions (WRITE-CONTACTS, ADD_VOICEMAIL, USE_SIP, BODY_SENSORS, RECEIVE_SMS and READ_SMS) that did not contribute significantly to malicious detection. MADFU uses the remaining 18 permissions to classify malicious apps.

### 4.4. Joint Probabilities Analysis

**Figure 6:**
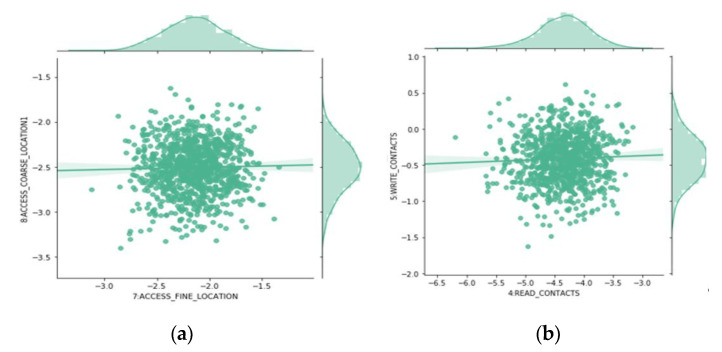
Joint Probability Distributions of β Values for Different Combinations of Permissions. ((**a**) β Values for ACCESS_FINE_LOCATION and ACCESS_COARSE_ LOCATION, (**b**) β Values for READ_CONTACTS and WRITE_ CONTACTS).

After analyzing the probability distribution of different model parameters in detail, we study their joint probabilities, in order to discover more interesting patterns. Scatters are used to represent different permissions relationship, for some highly relevant permission pairs such as ACCESS_FINE_LOCATION and ACCESS_COARSE_ LOCATION (Figure 6a). Most malicious apps use both permissions at the same time. However, most benign apps use one alone. We found similar relationships among several other pairs, such as: READ_CALL_LOG and WRITE_CALL_LOG. On the contrary, READ_CONTACTS and WRITE_ CONTACTS (Figure 6b) belong to the same group of dangerous permissions. However, malicious apps prefer to use one of them. Most benign apps use both.

### 4.5. Performance of Detection

In the following section, we evaluate the detection performance of our method with 5 machine learning classifiers (NB, BN, J48, RT and RF) on 1145 samples. The experimental results are shown in Table 1.

It can be seen from Table 1 that the FPR, accuracy and F-measure of the J48 classifier are 0.012, 95.5% and 94.7%. The AUC of the J48 reaches 0.944. The J48 classifier’s results (FPR, accuracy, F-measure and AUC) are all superior to other classifiers.

It can be seen from Table 1 that the accuracy of all the classifiers, except RF, exceeded 91%. RF has advantages for high-dimensional data, feature missing data and unbalanced data, but it may have unsatisfactory classification effect for small or low-dimensional data. Because MADFU only uses dangerous permissions, and the dimension is small, the classification effect is not ideal.

**Figure 7:**
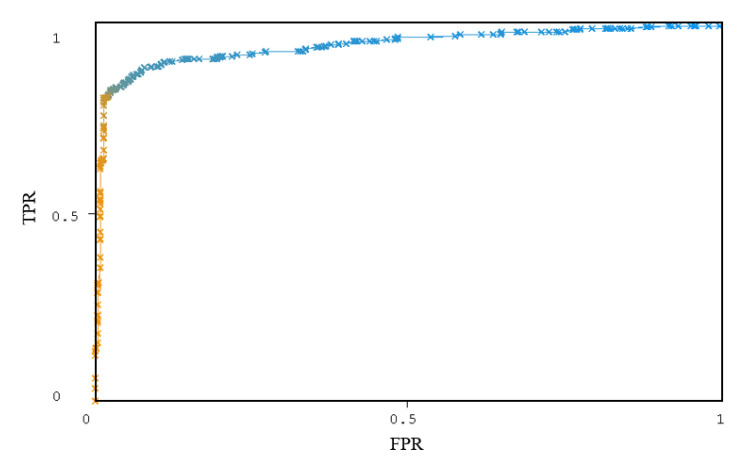
The ROC curves of J48.

The ROC curve of the J48 classifier is shown in Figure 7. From Figure 7, the curve is close to the upper left, and the area AUC under the curve is 0.944. According to AUC and ROC curves, the J48 classifier has better classification effect. Therefore, J48 is used in the MADFU for classification.

Through the analysis of 4.3, we removed 6 dangerous permissions that did not contribute significantly to Android app detection. We retained 18 dangerous permissions that contribute to malicious application detection for machine learning and classification. We used the J48 classifier after 10-fold cross validation; the results are shown in Table 2.

From Table 2, we can see that after 1145 samples are classified with 18 selected dangerous permissions, MADFU’s accuracy can reach 95.5%, F-measure can reach 94.7%, and FPR can be 1.2%. MADFU’s classify performance is higher than using 24 dangerous permissions, because the proposed approach uses fewer features in learning and classification.

As described before, we have collected 892 real-world datasets. We evaluate the app with MADFU. It can be obtained from Table 2, for unknown/new samples, that the MADFU’s accuracy can reach 92.7%, and FPR is 5.6%.

### 4.6. Compare with Other Approach

In this section, we compare MADFU with other state of-the-art malware detection approaches. We compare it with the method that only uses permissions features. SIGPID [28] is an approach that applies permission ranking to detection. We reimplemented their approach for comparison. Moreover, chi-square is a popular feature selection method [1]; so, in our paper, MADUF and chi-square methods are also compared. The comparison results are shown in Table 3.

SIGPID uses 22 significant permissions to classify different families of malware and benign apps. Compared with SIGPID, the F-measure of MADFU is 95.5%, and the SIGPID is 94.6%. SIGPID takes 1.5 times as long to learns and tests data as the method. The MADFU method has a higher F-measure and less training and learning time. Above all, MADFU performs better than the SIGPID method in this case. It can be seen from Table 3 that MADFU is superior to chi-square in accuracy and F-measure.

The accuracies of FEST [18] and FgDetector [19] were 98% and 98.15%, respectively. These are better than MADFU, at 95.5%. However, FEST is detected by 5 types (permission, API, action, IP and URL) of 398 typical features. FgDetector used the hardware components, requested permissions, app components, filtered intents, API calls and used permissions etc. for detection. MADFU only uses 18 dangerous permissions for analysis. So, MADFU has small feature dimension and high efficiency in learning and classification.

## 5. Discussion and Future Work

In this paper, we use the logistic regression function to model the Android app detection model and use the MCMC algorithm to sample the model parameters. During the analysis, we found that there are uncertainties in the features of Android apps’ detection. The uncertainty of these features affects the detection effect. MADFU solves the uncertainty in the detection process of dangerous permissions. The detection effect of MADFU is improved compared with the detection effect of directly using 24 dangerous permissions. In this paper, only 18 dangerous permissions, with the largest contributions to classification, are used for detection. Compared with other methods, MADFU learning and classification time and memory consumption are reduced.

Although there are discoveries revealed by these studies, there are also limitations. First, MADFU only uses dangerous permissions to classify Android apps, but some root-exploit apps do not have permission for use during the analysis. Therefore, it is impossible to classify such apps only by permissions. Second, during the analysis, some features with high correlation authority and negative correlation were found. These correlations may have a greater contribution to the classification of Android apps. Third, the majority of apps need to connect to a network to send and receive data, receive updates, and malware may send users’ personal data to instruction through network. Using monitoring network operations of apps on mobile devices is an effective way of catching the malicious behaviors of apps. We will carry out further research on these open issues.

## 6. Conclusions

In this paper, we present a new static detection technology called MADFU. Through the MCMC algorithm, MADFU calculates the uncertain value of the permission feature in the machine learning classification process. After the uncertainty analysis, 18 permission features are retained for machine learning classification. Compared with the method of directly using 24 dangerous permissions for classification, MADFU has a higher accuracy for the security detection of the Android app. MADFU’s accuracy can reach 95.5%. At the same time, MADFU has a better detection effect on unknown Android apps, and its accuracy can reach 92.7%. Compared with SIGPID, MADFU has a shorter learning and classification time, and has a higher accuracy.

## Figures and Tables

**Table 1:** Classification results of different classifiers.

Classifier	False Positive Rate (FPR)	Accuracy	F-Measure	Area under Curve (AUC)
NB	0.083	91.5%	88.3%	0.83
BN	0.088	91.1%	90.3%	0.901
J48	0.012	95.5%	94.7%	0.944
RT	0.081	91.8%	91.4%	0.89
RF	0.47	69.5%	44.5%	0.451

**Table 2:** MADFU’s classify performance for different datasets.

Datasets Number	Method	FPR	Accuracy	F-Measure
1145 (589 begin/556 malicious)	24 Dangerous Permissions	0.057	88.7%	87.5%
	MADFU	0.012	95.5%	94.7%
892 (469 begin/423 malicious)	MADFU	0.056	92.7%	91.3%

**Table 3:** MADFU compared with other approach.

Method	Accuracy	F-Measure	Learning and Classification Times(s)
SIGPID	94.6%	91.6%	4.5
Chi-Square	93.1%	91.2%	3.1
MADFU	95.5%	94.7%	3

**Figure A1:**
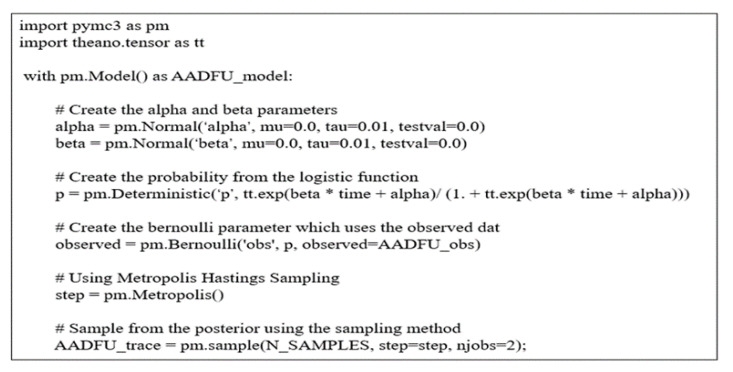
AADUF Model represented in PyMC3.

## Appendix A

The evaluation terms of the classification algorithm are shown in Table A1. The benign samples are positive and the malicious samples are negative.

**Table A1:** The evaluation terms of the classification algorithm.

	Positive (Predicted)	Negative (Predicted)
Positive (Actual)	TP (True Positive)	FN (False Negative)
Negative (Actual)	FP (False Positive)	TN (True Negative)

Other evaluation indicators are defined as follows.

FPR represents the false positives rate that is incorrectly identified as positive. FPR is defined as follows:

*FPR* = *FP*/(*FP* + *TN*) (A1)

Accuracy is the percentage of tuples that are correctly classified by the classifier. Accuracy is defined as follows:

*Accuracy* = (*TP* + *TN*)/(*TP* + *FP* + *TN* + *FN*) (A2)

F-Measure represents the harmonic mean of *Precision* = *TP*/(*TP* + *FP*) and *Recall* = *TP*/(*TP* + *FN*). F-Measure is defined as follows:

*F-Measure* = (*2* × *Precision* × *Recall*)/(*Precision* + *Recall*) (A3)

ROC curve refers to the lines drawn by taking *FPR* as the X-coordinate and TPR as the Y-coordinate.

Area under curve (AUC) is defined as the area under ROC curve. The ROC curve does not clearly indicate which classifiers perform better. However, AUC can better evaluate the classifier. The greater the AUC, the better the classifier.

## Appendix B

The PyMC3 code is shown in Figure A1.
